# Epidemiological and clinical characteristics of road traffic crashes related thoracic traumas: analysis of 5095 hospitalized chest injury patients

**DOI:** 10.1186/s13019-021-01599-4

**Published:** 2021-08-04

**Authors:** Shengcao Zhang, Xiangzhi Xiao, Jian Wang, Chengkai Hu, Qiancheng Du, Zheng Fu, Wei Cai, Zhilong Zhang, Hao Chen

**Affiliations:** 1 Department of Thoracic Surgery, Zhongshan Hospital Qingpu Branch, Fudan University, 1158 East Parkway, Shanghai, 201700 China; 2 Department of Thoracic Surgery, Zhongshan Hospital Xuhui Branch, Fudan University, 966 Huaihai Road, Shanghai, 200032 China

**Keywords:** Thoracic trauma, Chest injury, Road traffic crashes, Rib fractures, Mechanical ventilation, Surgery, Lung contusion

## Abstract

**Background:**

Road traffic crashes related (RTCR) chest traumas remain important global public health challenge. The impact of boosting market of automobile vehicles in China during last decade on thoracic injury needs to be defined. This study aimed to review and analyze the demographic and clinical characteristics of RTCR thoracic injuries in China.

**Methods:**

Clinical records of patients with thoracic trauma admitted to thoracic surgery department between January 2003 and June 2020 were retrospectively retrieved and reviewed. Patients’ profiles and clinical characteristics were comparatively analyzed between road traffic crashes caused injury and other injury mechanisms, and in RTCR chest trauma patients before January 2011 (2003 group), and after January 2011 (2011 group), when is considered as the beginning year of Chinese household vehicle era.

**Results:**

The study included 5095 thoracic trauma patients with mean age of 50.2 years, of whom 79.4% were male. Most of the patients (70.3%, *n* = 3583) had rib fractures. Associated injuries were present in 52.0% of the patients, of them 78.5% (*n* = 2080) were extremity fractures. Road traffic crashes accounted for 41.4% (*n* = 2108) of the injuries, most of them (98.0%) were related to motor vehicles. In comparison with other chest trauma mechanisms, RTCR chest injuries affected females and older males more frequently, with a higher incidence of rib fractures and sternum fractures, and higher injury severity scores (ISS) (all *p* < 0.05). Surgeries were required in 1495 (70.9%) patients of the RRTCR chest traumas, while the majority of non-RTCR thoracic injuries were managed conservatively or with tube thoracostomy (30.2%,* n* = 901). RTCR chest traumas caused longer hospital stay (13.0 ± 9.6 days vs. 11.8 ± 7.4 days, *p* = 0.001), higher ICU usage (30.7% vs. 19.6%, *p* = 0.001), higher rate of ventilator support (12.9% vs. 7.5%, *p* = 0.001), and higher mortalities (3.8% vs. 1.6%, *p* = 0.005) than that of non-RTRA chest injuries. For RTCR patients, when compared with 2003 group, 2011 group had similar patterns in terms of accident category, associated injury and treatment. However, 2011 group had more females (38.5% vs. 18.0%, *p* = 0.001) and older males (50.6 ± 9.7 vs. 47.9 ± 17.2, *p* = 0.001), with a higher ISS (18.3 ± 10.2 vs. 17.1 ± 8.9, *p* = 0.004), and fewer were managed with chest tubes (25.0% vs. 29.2%, *p* = 0.031). Clinical outcomes were not significantly different between the groups in terms of hospital length of stay, intensive care unit (ICU) usage, ICU length of stay, duration of ventilator hours and mortality. However, the 2011 group had more patients requiring ventilator support (14.4% vs. 10.6%, *p* = 0.011).

**Conclusions:**

Road traffic crashes remain to be the major etiology of thoracic injuries in China, which usually affects middle-aged males, causing rib fractures with concomitant injuries frequently occurring to other organ systems. Treatments mainly include tube thoracotomy and surgical procedures. Although the clinical characteristics and outcomes of traffic accident related chest traumas are largely unchanged in spite of the rapid increasing numbers of motor vehicles, variations in the pattern of injuries by gender, age, injury severity and ventilator usage may still provide important information for targeted management.

## Background

Road traffic injuries remain a major public health challenge globally. According to the World Health Organization (WHO), approximately 1.35 million people die and 20–50 million injured each year as a result of road traffic crashes. Road traffic crashes are the eighth leading cause of death for people of all ages, and the number one killer of people aged 5–29 years. Economic burden associated with traffic injuries are also substantial, costing most countries 3% of their gross domestic product. Furthermore, 93% of the fatalities on the roads occur in low- and middle-income countries, the risk of dying in a road traffic crash is more than 3 times higher in developing countries than in developed countries [1].

It is known that thoracic injuries accounted for approximately 25% of all traumas associated mortalities, as thorax hosts vital organs such as heart, lung, great vessels and esophagus [2, 3]. Similar to many other nations, road traffic crashes is the top one injury mechanism for thoracic traumas in China [4–8]. According to Chinese authorities, the car parc of year 2003 is 24 million. In 2011 the number of registered motor vehicles reached 106 million. This number has been more than tripled since then, reached an all-time high of 372 million by year 2020 [9]. While a tremendous increase in numbers of registered automobiles has been seen for the past decade, the epidemiology of road traffic crashes related (RTCR) thoracic traumas remains understudied. Per WHO’s estimation, road traffic crashes deaths for China were 256,180 in year 2016 with a mortality rate of 18.2 per 100,000 populations [1]. However, the paucity of literature on epidemiological and clinical characteristics of RTCR chest injuries in China, the biggest developing country, and the largest auto manufacturing and consuming country in the world, would withhold for optimal policy-making and targeted preventive strategies.

Therefore, the present study is aimed to review and describe the demographic and clinical characteristics of RTCR thoracic injuries in Shanghai, one of the most densely populated cities of China. The study will also analyze and define the impact of such a rocket speed growing number of motor vehicles in the past decade on thoracic injury in a tertiary university hospital serving more than one million residents.

## Methods

### Study population

Consecutive patients with thoracic trauma who admitted to the department of thoracic surgery between January 2003 and June 2020 were included. Clinical records including medical images were retrieved from hospital clinical information center electronically, and reviewed in terms of age, gender, etiology, injury type, injury severity, treatment, length of hospital stay, and clinical outcomes. Patients treated only in an emergency or outpatient setting or transferred to another institution were excluded.

The patients’ demographic profiles, clinical characteristics and outcomes were analyzed and compared between traffic accident caused injuries and other injury mechanisms, and in RTCR patients before January 2011 (2003 group) and after January 2011 (2011 group), as year 2011 is considered as the beginning year of Chinese household automobile era. In response to the dynamic medical demands, a dedicated thoracic trauma team consisting of thoracic surgeons, trauma surgeons, emergency physicians, anesthetists, respiratory and radiology specialists was implemented in January 2012 [8]. After that the protocol of triaging, diagnosis, assessment and therapy for thoracic injuries were optimized and standardized, as described below.

In brief, after admission, patients were evaluated by systemic physical examination, chest computed tomography (CT), electrocardiography and blood tests. Those of whom had other severe injuries, respiratory difficulties, and hemodynamic instabilities were managed in intensive care unit (ICU) and used mechanical ventilator if necessary. Patients were also evaluated in terms of coexisting pathologies and followed up closely by concerned specialists. Tube thoracostomy was performed in patients with pneumothorax or hemopneumothorax per surgeon’s discretion. General in-hospital surveillance included monitoring of respiration, heart rate, arterial blood pressure, central venous pressure, urine output, thoracic drainage, arterial gas analyses, etc.

Non-operative treatment basically consisted of oxygen inhalation, mechanical ventilation if indicated, pain control (oral or intravenous nonsteroidal anti-inflammatory analgesics, and loco-regional anesthesia such as intercostal nerve blocks and thoracic epidural catheters), bronchodilators and mucolytics, and antibiotics when indicated. Emergent or elective surgeries were carried out for patients who failed early, optimal conservative management, with a flail chest (defined as 3 or more consecutive ribs have multiple fractures within each rib, with clinical signs of paradoxical chest wall movement), or multiple, severe displaced fractures requiring video-assisted thoracoscopic surgery or thoracotomy.

All patients were managed by a same group of thoracic surgeons, and had follow-up visits at 2 and 4 weeks after discharge, with chest CT scans upon the visit.

The study protocol was approved by the Institutional Review Board of Zhongshan Hospital Qingpu Branch, Fudan University. Patients consent was waived.

### Statistical analysis

Data were presented as number, frequencies, percentages, or mean ± standard deviation (SD) whenever appropriate. Differences in categorical variables between groups were compared using the chi-squared test. Numbers of patient admitted by year were examined for correlation between groups by calculating the Spearman correlation coefficient. Differences in continuous variables between groups were tested for normality using Shapiro–Wilk normality test, and compared using the student’s unpaired t test (for parametric distribution) or the Mann–Whitney U-test (for nonparametric distribution), as appropriate. Data analysis was carried out using the Statistical Package for Social Sciences version 20 for Mac (SPSS Inc., Chicago, IL). Two sided *p* values of less than 0.05 were considered statistically significant.

## Results

### Patient characteristics—all cause chest traumas

The study included a total of 5095 patients with chest injury during the study period. There were 4045 (79.4%) male and 1050 (20.6%) female patients whose ages ranged from 13 to 103 years with an average age of 50.2 ± 14.2 years (male, 48.6 ± 14.5 years; female, 55.9 ± 13.3 years). Chest trauma was observed most frequently in patients aged between 31 and 60 years (*n* = 2816, 55.3%). The most common injury mechanism was road traffic crashes (*n* = 2108, 41.4%), most of them (*n* = 2065, 98.0%) were related to motor vehicles, followed by slip down (*n* = 1432, 28.1%), falls (*n* = 678, 13.3%), assault (*n* = 459, 9.0%), and industrial accident (*n* = 418, 8.2%). When classified according to the injury type, the vast majority patients had rib fractures (*n* = 3583, 70.3%). Other injury types included pneumothorax (*n* = 1019, 20.0%), hemopneumothorax (*n* = 247, 4.8%), lung contusion (*n* = 121, 2.4%), sternum fracture (*n* = 105, 2.1%) and hemothorax (*n* = 20, 0.4%).

Concomitant injuries were present in 52.0% of the patients (*n* = 2649), most of them had extremity fractures (*n* = 2080, 78.5%), followed by abdominal injuries (*n* = 232, 8.8%), clavicle fractures (*n* = 210, 7.9%), head and spinal injuries (*n* = 96, 3.6%) and facial bone fractures (*n* = 31, 1.2%), with an average injury severity score (ISS) of 15.4 ± 10.1. Table 1 summarizes the demographic and clinical characteristics of these 5095 thoracic trauma patients.

**Table 1:** The demographic and clinical characteristics of 5095 thoracic trauma patients

	Number or mean	Percent	SD
Total	5095	100.0	
Gender			
Male	4045	79.4	
Female	1050	20.6	
Age (year)	50.2		14.2
Male	48.6		14.5
Female	55.9		13.3
< 31	828	16.3	
31–60	2816	55.3	
> 61	1451	28.5	
Injury mechanism			
Traffic accident	2108	41.4	
Slip down	1432	28.1	
Falls	678	13.3	
Assault	459	9.0	
Industrial accident	418	8.2	
Injury type			
Rib fracture	3583	70.3	
Pneumothorax	1019	20.0	
Hemopneumothorax	247	4.8	
Lung contusion	121	2.4	
Sternum fracture	105	2.1	
Hemothorax	41	0.8	
Associated injury	2649	52.0	
Extremity fracture	2080	78.5	
Abdominal	232	8.8	
Clavicle fracture	210	7.9	
Head and spinal	96	3.6	
Facial	31	1.2	
ISS	15.4		10.1

Categorical data are presented as number and percent; continuous data are presented as mean ± SD

*SD* standard deviation, *ISS* injury severity score

### Patient characteristics—traffic accident related chest traumas versus non-traffic accident related chest traumas

The RTCR chest injury patient’s profiles in comparison with non-traffic accident related thoracic traumas are shown in Table 2. Road traffic crashes ranked the number 1 injury mechanism, accounted for 41.4% (2108 out of 5095) of all chest injuries. Other injury mechanisms, such as slip, falls, assault, and industrial accident, were responsible for the rest patients (*n* = 2987, 58.6%). Approximately 30% (*n* = 632) of the RTCR chest injuries affected females, whereas 86% (*n* = 2569) of the non-RTCR chest trauma patients were male (*p* = 0.001). Comparing to non-RTCR patients, male RTCR patients were older (49.3 ± 13.3 years vs. 48.2 ± 15.1 years, *p* = 0.016).

**Table 2:** Clinical characteristics of road traffic crashes related thoracic injuries in comparison with non-road traffic crashes related chest traumas

	RTCR chest trauma (*n* = 2108)	Non-RTCR chest trauma (*n* = 2987)	*p*
Gender (n, %)			
Male	1476, 70.0%	2569, 86.0%	**0.001**
Female	632, 30.0%	418, 14.0%	
Age (year)	51.1 ± 13.5	49.6 ± 14.7	**0.001**
Male	49.3 ± 13.3	48.2 ± 15.1	**0.016**
Female	55.3 ± 12.4	56.8 ± 14.7	0.086
Injury type (n, %)			
Rib fracture	1571, 74.5%	2012, 67.4%	**0.001**
Pneumothorax	351, 16.7%	668, 22.4%	**0.001**
Hemopneumothorax	105, 5.0%	142, 4.8%	0.710
Lung contusion	45, 2.1%	76, 2.5%	0.344
Sternum fracture	65, 3.1%	40, 1.3%	**0.001**
Hemothorax	21, 1.0%	20, 0.7%	0.199
Associate injury (n, %)	1177, 55.8%	1472, 49.3%	**0.001**
Extremity fracture	912, 77.5%	1168, 79.3%	0.246
Abdominal	111, 9.4%	121, 8.2%	0.273
Clavicle fracture	94, 8.0%	116, 7.9%	0.920
Head and spinal	48, 4.1%	48, 3.3%	0.263
Facial	14, 1.2%	17, 1.2%	0.934
ISS	17.7 ± 9.4	13.6 ± 10.5	**0.001**

*p* < 0.05 are given in bold

Categorical data are presented as number and percent; continuous data are presented as mean ± SD

*SD* standard deviation, *ISS* injury severity score, *RTCR* road traffic crashes related

The incidences of rib fracture and sternum fracture were significantly higher in RTCR patients than that of non-RTCR patients (74.5% vs. 67.4%, and 3.1% vs. 1.3%, respectively, all *p* = 0.001). On the other hand, non-RTCR patients had a higher rate of pneumothorax compared to RTCR patients (22.4% vs. 16.7%, *p* = 0.001). Other injury types, such as hemopneumothorax, lung contusion, and hemothorax, were not remarkably different between RTCR and non-RTCR patients (all *p* > 0.05). Concomitant injuries occurred more frequently in RTCR patients, compared to non-RTCR patients (55.8% vs. 49.3%, *p* = 0.001), yet no differences were found in specific organ systems, such as extremity, abdomen, clavicle, head and spinal, and facial bone (all *p* > 0.05). RTCR chest trauma patients had a significant higher ISS score than that of non-RTCR patients (17.7 ± 9.4 vs. 13.6 ± 10.5, *p* = 0.001).

### Patient characteristics—traffic accident related chest traumas variations during study period

**Fig. 1:**
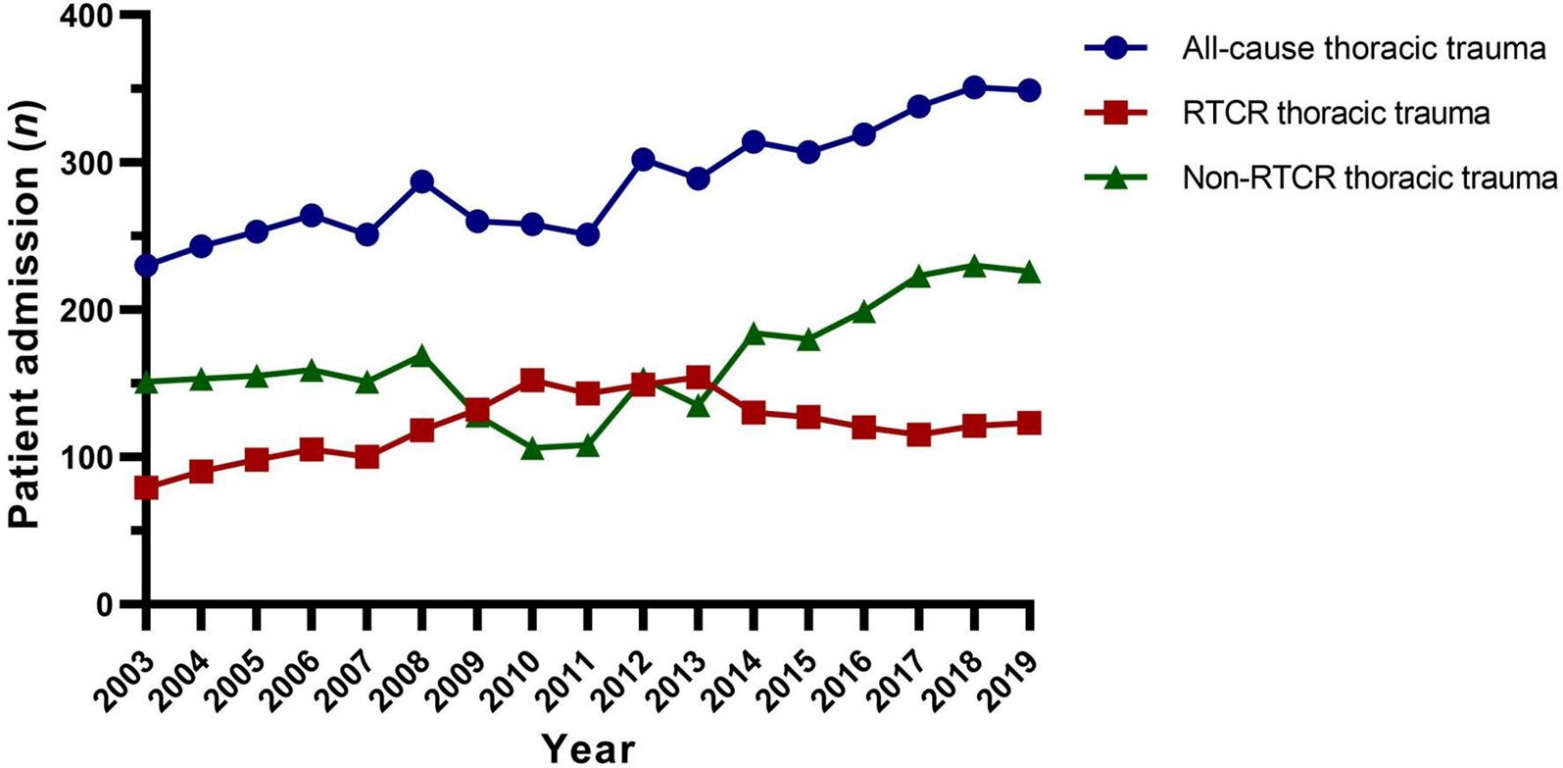
Annual numbers of thoracic trauma patient admitted to the Department of Thoracic Surgery, from Jan 2003 to Dec 2019. *RTCR* road traffic crashes related

Figure 1 shows the numbers of thoracic injury patients admitted to thoracic surgery department each year from 2003 to 2019. A trend was seen that the annual numbers of admitted all-cause chest trauma patient were gradually increased during the study period. Numbers of annual admitted non-RTCR patient mirrored to this trend. Nonetheless, after rising for years and reaching a plateau between 2010 and 2013, the numbers of admitted RTCR patient decreased in 2014 and remained stationary afterwards, seemed irresponsive to the gradual growth of all-cause thoracic injured patients.

Spearman correlation coefficient test confirmed these findings that the numbers of admitted all-cause thoracic trauma patient by year correlated with that of non-RTCR thoracic trauma patient (*r* = 0.791, *p* < 0.001), but not with that of RTCR group (*r* = 0.329, *p* = 0.197), nor between groups of RTCR and non-RTCR (*r* =  − 0.280, *p* = 0.275).

Table 3 summarized the demographic and clinical characteristics of RTCR thoracic trauma patients in different study periods. There were 2046 and 3049 patients admitted before- and after- January 2011, respectively. Eight hundred and seventy four chest trauma cases were related to traffic accident in the 2003 group, which is numerically higher than that of the 2011 group (*n* = 1234), yet no statistical differences were reached (42.7% vs. 40.5%, *p* = 0.111). The majority casualties were male in both the 2003 and 2011 groups (82.0% and 61.5%, respectively), however more female patients were found in the 2011 group (18.0% vs. 38.5%, *p* = 0.001). When compared to 2003 group for patient’s age, a trend of older male victims was seen in 2011 group (47.9 ± 17.2 years vs. 50.6 ± 9.7 years, *p* = 0.001). The traffic accident category component remained stable, of which motor vehicles were responsible for most of the chest injuries throughout the study period, although automobiles related chest injuries were numerically higher in the 2011 group, in comparison with 2003 group (98.2% vs. 97.6%, *p* = 0.321).

**Table 3:** Road traffic crashes related thoracic injury patient characteristics comparison before and after Jan 2011

	2003 group	2011 group	*p*
Total chest trauma (n)	2046	3049	
RTCR chest trauma (n, %)	874, 42.7%	1234, 40.5%	0.111
Gender (n, %)			
Male	717, 82.0%	759, 61.5%	**0.001**
Female	157, 18.0%	475, 38.5%	
Age (year)	49.2 ± 17.1	52.4 ± 10.9	**0.001**
Male	47.9 ± 17.2	50.6 ± 9.7	**0.001**
Female	55.1 ± 16.5	55.3 ± 11.0	0.887
Accident category (n, %)			
Motor	853, 97.6%	1212, 98.2%	0.321
Non-motor	21, 2.4%	22, 1.8%	
Injury type (n, %)			
Rib fracture	611, 69.9%	910, 73.7%	0.053
Pneumothorax	168, 19.2%	183, 14.8%	**0.001**
Hemopneumothorax	43, 4.9%	62, 5.0%	0.914
Lung contusion	19, 2.2%	26, 2.1%	0.917
Sternum fracture	24, 2.7%	41, 3.3%	0.451
Hemothorax	9, 1.0%	12, 1.0%	0.896
Associate injury (n, %)	478, 54.7%	699, 56.6%	0.373
Extremity fracture	377, 78.9%	535, 76.5%	0.347
Abdominal	42, 8.8%	69, 9.9%	0.532
Clavicle fracture	37, 7.7%	55, 7.9%	0.936
Head and spinal	17, 3.6%	31, 4.4%	0.454
Facial	5, 1.0%	9, 1.3%	0.707
ISS	17.1 ± 8.9	18.3 ± 10.2	**0.004**

*p* < 0.05 are given in bold

Categorical data are presented as number and percent; continuous data are presented as mean ± SD

*SD* standard deviation, *ISS* injury severity score, *RTCR* road traffic crashes related

In regard of injury type, both the groups shared similar pattern, most of them were rib fractures, except that the 2003 group had more hemothorax patients as compared to that of 2011 group (19.2% vs. 14.8%, *p* = 0.001). The incidents of associated injuries were not significantly different between the groups (54.7% vs. 56.6%, *p* = 0.373). The constituents of the associate injury were also similar between the groups, the vast majority were extremity fractures (78.9% vs. 76.5%, *p* = 0.347). However, the ISS scores of 2011 group were remarkably higher than that of the 2003 group (18.3 ± 10.2 vs. 17.1 ± 8.9, *p* = 0.004).

### Treatment and clinical outcomes—road traffic crashes related versus non traffic accident related

The treatments and clinical outcomes were compared between RTCR chest trauma patients and thoracic trauma patients with other injury mechanisms. As shown in Table 4, less RTCR chest trauma patients were managed with chest tubes alone than non-RTCR patients did (26.7% vs. 30.2%, *p* = 0.007). On the other hand, emergent or elective surgeries were more frequently performed in RTCR patients than in non-RTCR patients (70.9% vs. 24.1%, *p* = 0.001). Major clinical outcome parameters were significantly less favorable in RTCR group than that of non-RTCR group, with a longer length of hospital stay (13.0 ± 9.6 days vs. 11.8 ± 7.4 days, *p* = 0.001), a higher ICU usage (30.7% vs. 19.6%, *p* = 0.001), a higher rate of mechanical ventilator support (12.9% vs. 7.5%, *p* = 0.001), and a higher death rate (3.8% vs. 1.6%, *p* = 0.005). However, the two groups of patients shared similar ICU length of stay (4.8 ± 1.9 days vs. 4.6 ± 1.7 days, *p* = 0.191), and duration of ventilator support (135.6 ± 18.2 h vs. 132.4 ± 19.3 h, *p* = 0.058).

**Table 4:** Treatment and clinical outcomes of traffic crashes related thoracic injury patients in comparison with non-traffic crashes related chest traumas

	RTCR chest trauma (*n* = 2108)	Non-RTCR chest trauma (*n* = 2987)	*p*
Treatment (n, %)			
Chest tube	563, 26.7%	901, 30.2%	**0.007**
Surgery	1495, 70.9%	720, 24.1%	**0.001**
Outcome			
Hospital stay (day)	13.0 ± 9.6	11.8 ± 7.4	**0.001**
ICU			
ICU usage (n, %)	648, 30.7%	586, 19.6%	**0.001**
ICU duration (day)	4.8 ± 1.9	4.6 ± 1.7	0.191
Ventilator			
Ventilator usage (n, %)	271, 12.9%	225, 7.5%	**0.001**
Ventilator duration (h)	135.6 ± 18.2	132.4 ± 19.3	0.058
Mortality (n, %)	81, 3.8%	47, 1.6%	**0.005**

*p* < 0.05 are given in bold

Categorical data are presented as number and percent; continuous data are presented as mean ± SD

*SD* standard deviation, *RTCR* road traffic crashes related

### Treatment and clinical outcomes—traffic accident related chest traumas before and after Jan 2011

Road traffic crashes related thoracic injury patients were also compared before and after January 2011, the year when national car parc exceeded 100 million. As shown in Table 5, more patients received tube thoracostomy alone in 2003 group as compared to 2011 group (29.2% vs. 25.0%, *p* = 0.031). Numerically more patients of the 2011 group underwent surgical procedures than 2003 group did, however no statistical significance was reached (72.3% vs. 69.0%, *p* = 0. 101). The most common surgical procedure was for rib fracture in both groups, no difference was found between them (43.8% vs. 42.6%, *p* = 0.651), nor for other surgery types, such as hemopneumothorax (33.8% vs. 36.1%, *p* = 0.368), pulmonary laceration (17.4% vs. 14.6%, *p* = 0.139), diaphragm rupture (2.2% vs. 3.1%, *p* = 0.254), esophageal rupture (1.8% vs. 2.4%, *p* = 0.487), and heart contusion (1.0% vs. 1.2%, *p* = 0.670).

**Table 5:** Treatment and clinical outcomes of road traffic crashes related thoracic injury patients before and after Jan 2011

	2003 group (*n* = 874)	2011 group (*n* = 1234)	*p*
Treatment (n, %)			
Chest tube	255, 29.2%	308, 25.0%	**0.031**
Surgery	603, 69.0%	892, 72.3%	0.101
Rib fracture	264, 43.8%	380, 42.6%	0.651
Hemopneumothorax	204, 33.8%	322, 36.1%	0.368
Pulmonary laceration	105, 17.4%	130, 14.6%	0.139
Diaphragm rupture	13, 2.2%	28, 3.1%	0.254
Esophageal rupture	11, 1.8%	21, 2.4%	0.487
Heart contusion	6, 1.0%	11, 1.2%	0.670
Outcome			
Hospital stay (day)	13.2 ± 9.1	12.9 ± 8.7	0.444
ICU			
ICU usage (n, %)	253, 28.9%	396, 32.1%	0.123
ICU duration (day)	4.7 ± 1.5	4.9 ± 2.1	0.158
Ventilator			
Ventilator usage (n, %)	93, 10.6%	178, 14.4%	**0.011**
Ventilator duration (h)	134.2 ± 19.1	136.3 ± 17.5	0.364
Mortality (n, %)	32, 3.7%	49, 4.0%	0.723
Hemorrhagic shock	16, 50.0%	21, 42.9%	0.528
Respiratory failure	10, 31.2%	18, 36.7%	0.612
Cardiac tamponade	6, 18.8%	10, 20.4%	0.855

*p* < 0.05 are given in bold

Categorical data are presented as number and percent; continuous data are presented as mean ± SD

*SD* standard deviation

The hospital length of stay was comparable between 2003 and 2011 group (13.2 ± 9.1 days vs. 12.9 ± 8.7 days, *p* = 0.444). ICU treatment was necessary for 253 (28.9%) patients of the 2003 group, and 396 (32.1%) patients of the 2011 group, respectively, with no statistical difference between them (*p* = 0.123). No difference was found for the average ICU stay between the groups either (4.7 ± 1.5 vs. 4.9 ± 2.1, *p* = 0.158). The 2011 group had much more patients requiring mechanical ventilator support than the 2003 group did (14.4% vs. 10.6%, *p* = 0.011). However, no difference was found for the average duration of ventilator support between the groups (136.3 ± 17.5 h vs. 134.2 ± 19.1 h, *p* = 0.364).

Although the mortality rate of 2011 group is numerically higher than that of 2003 group, no statistical difference was found (4.0% vs. 3.7%, *p* = 0.723). Specifically, patients died of hemorrhagic shock, respiratory failure and cardiac tamponade were all comparable between the groups (50.0% vs. 42.9%, *p* = 0.528; 31.2% vs. 36.7%, *p* = 0.612; 18.8% vs. 20.4%, *p* = 0.855, respectively).

## Discussion

To our knowledge, this is the first study on China’s RTCR thoracic traumas during an extraordinary timeframe, a period full of rapid, dynamic changes of road users, traffic safety regulations, and targeted therapeutic protocols. As the biggest developing country, and the largest motor vehicles manufacturing and consuming nation, China’s car parc has been expanding in a rocket speed for over a decade. It is interesting and important to define the roles of such a fast increasing numbers of automobiles in the epidemiologic and clinical features of thoracic traumas, whereas related literatures are scarce.

The present study firstly investigated the impacts of road traffic crashes on thoracic traumas. The demographic and clinical profiles of patients of RTCR thoracic traumas were reviewed and analyzed. In line with many other authors [3–6, 10], our data demonstrated that road traffic crashes is the number one etiology for chest injuries, costs more healthcare resources, and causes more comorbidities and fatalities than any other injury mechanisms. Specifically, females and older males are more vulnerable to road traffic crashes, fractures of rib and sternum and co-existing injuries are more frequent in RTCR chest injured patients. Furthermore, chest injuries involved in road traffic crashes are usually more severe, often necessitate surgical interventions, intensive care and mechanical ventilator support. These findings highlight the significance of public efforts on road safety awareness, associated policy making and enforcement, road design, and other targeted preventions [11–13].

The impacts of rapid increasing car parc were then examined by comparing the demographic and clinical characteristics of RTCR chest trauma patients in different periods, as before- or after- 2011, which is the year when the numbers of national registered automobiles exceeded 100 million, and kept climbing till now. We found that RTCR thoracic trauma patients admitted after year 2011 consisted of more females and older males, a speculated reason for this difference is that with the recent continually economic growth, more of these groups can now afford to buy automobiles, with more severe ISS score than that of RTCR patients prior to 2011. These changes may call for proper management protocol adjustment such as closer collaboration with geriatricians for elderly patients or with obstetricians for pregnant women. It has been reasonably supposed that more automobiles on the road lead to more road traffic crashes and more related morbidities and mortalities. However, our results indicated that (1) changes of the numbers of admitted RTCR chest trauma patient by year didn’t positively correlate with that of all-cause thoracic injuries; (2) the clinical portraits of RTCR thoracic traumas remain largely unchanged despite of the remarkable variations throughout the study period. Both study groups received similar surgical therapies, and their major clinical outcomes were comparable, in terms of either length of hospital stay, ICU usage, ICU length of stay, duration of on ventilator, or mortalities, except that patients admitted after year 2011 received less tube thoracotomy, and more of them needed mechanical ventilator support, which highlight the need to review the current healthcare resources allocation in response of such trend.

Although candidly the current status of China’s road safety is not as good as that of many high income countries, it is undeniable that conditions have been keep improving. Similar to other nations, credits would be given to economic growth, and authorities efforts for continuously promoting public safety awareness, enforcing the use of helmets and seat-belts, combatting speeding and drunk driving, and advocating for safer vehicles over the years [12, 14, 15]. Furthermore, newly instituted regional trauma centers with standardized, optimized managing protocol and dedicated task forces are also playing key roles [8, 16]. Yet no reductions in the number of RTCR chest injury deaths have been observed throughout the study period, which underlines the importance of keep strengthening strategies to mitigate key risk factors and to improve road safety [17–21].

The current study has both strengths and limitations. This is the one of the largest investigations on RTCR thoracic traumas, and to our knowledge the first examination of the impacts of car parc on RTCR thoracic traumas in China. Despite the known associated shortcomings inherent to the retrospective and observational design of the study, its retrospective nature can offer an epidemiologic picture of RTCR chest injuries. Besides, as currently there is no national trauma registry or clinical practice guideline for the management of chest traumas in China, different centers possess different clinical data and management protocols. This might have made it challenging for comparisons with other investigators. Furthermore, RTCR chest traumas may not adequately represented in the current study, as only those admitted to the department of thoracic surgery were investigated, others treated in the emergency department, outpatient department, or triaged or referred to elsewhere were not included. Lastly, this investigation only represents a single institution experience, cannot be generalized to the whole nation, and might not be able to detect other clinically meaningful differences. Nationwide multi-center prospective studies are needed to validate the results shown herein. Despite these limitations, the present study does provide valuable information on the epidemiologic and clinical profiles of RTCR chest injured patients of China, and the effects of motor vehicles boom on RTCR thoracic traumas, that could help to facilitate better targeted strategies.

## Conclusions

In summary, this large-scale retrospective investigation shows that road traffic crashes remain to be the major cause of thoracic injuries in China, leading to significant damage than any other injury mechanisms. Middle-aged males are most often liable, with rib fractures and associated injuries to other organ systems occuring frequently. Management regimen basically consists of tube thoracotomy and surgical operations. Moreover, the automobiles boom doesn’t remarkably impact the clinical characteristics and outcomes of RTCR thoracic traumas. However, detected changes of RTCR chest trauma patient profiles still provide important information for targeted evaluation, prevention, and management.

## Data Availability

The datasets used and analysed during the current study are available from the corresponding author on reasonable request.

## Abbreviations

RTCR: Road traffic crashes related
WHO: World Health Organization
ISS: Injury severity score
ICU: Intensive care unit
CT: Computed tomography
